# Diagnostic Performance of the International Academy of Cytology Yokohama System for Reporting Breast Fine-Needle Aspirates: A Cytology-Histopathology Correlation Study From Central India

**DOI:** 10.7759/cureus.113490

**Published:** 2026-07-28

**Authors:** Aditi Rathore, Archana Shrivastava, Maneesh Sulya

**Affiliations:** 1 Department of Pathology, Gandhi Medical College, Bhopal, IND

**Keywords:** breast cytology, diagnostic accuracy, fnac breast, histopathological correlation, risk of malignancy index (rmi), yokohama system

## Abstract

Background

Fine-needle aspiration cytology (FNAC) remains a rapid, minimally invasive, and cost-effective diagnostic method for breast lesions. The International Academy of Cytology (IAC) Yokohama System standardizes breast FNAC reporting into five diagnostic categories linked to risk of malignancy (ROM).

Objective

The objective of this study was to categorize breast fine-needle aspirates using the IAC Yokohama System and evaluate its diagnostic accuracy by correlation with histopathology.

Methods

This observational diagnostic accuracy study included 105 patients who underwent breast FNAC at the Department of Pathology, Gandhi Medical College and Hamidia Hospital, Bhopal. Aspirates were classified as C1 inadequate, C2 benign, C3 atypical, C4 suspicious for malignancy, or C5 malignant. Histopathology obtained during routine clinical management, rather than mandated by the study protocol, was used as the reference standard wherever available. Scenario A grouped C1-C2 as cytology-negative and C3-C5 as cytology-positive, whereas Scenario B grouped C1-C3 as cytology-negative and C4-C5 as cytology-positive. Two-sided 95% confidence intervals for category-specific ROM and binary diagnostic-performance estimates were calculated using the Clopper-Pearson exact method. The area under the curve (AUC) with its confidence interval was estimated using the DeLong method.

Results

The cohort consisted of 99 female cases and six male cases. The Yokohama distribution was C1: five cases, C2: 69 cases, C3: 10 cases, C4: three cases and C5: 18 cases. Histopathology was available in 100 cases. ROM was 0.0% (95% CI 0.0-84.2) for C1, 1.4% (95% CI 0.04-7.8) for C2, 40.0% (95% CI 12.2-73.8) for C3, 100.0% (95% CI 29.2-100.0) for C4 and 100.0% (95% CI 79.4-100.0) for C5. The primary diagnostic analysis yielded 23 true-positive, 70 true-negative, six false-positive and one false-negative results. Sensitivity was 95.8% (95% CI 78.9-99.9), specificity was 92.1% (95% CI 83.6-97.0), positive predictive value (PPV) was 79.3% (95% CI 60.3-92.0), negative predictive value (NPV) was 98.6% (95% CI 92.4-100.0), and overall accuracy was 93.0% (95% CI 86.1-97.1). Ordinal receiver operating characteristic (ROC) analysis yielded an AUC of 0.971 (95% CI 0.928-1.000).

Conclusion

The IAC Yokohama System provided clinically meaningful risk stratification and strong diagnostic performance in breast FNAC. The C3 atypical category remained the principal grey-zone group. C4 and C5 showed high observed ROM; however, the C4 estimate was based on only three cases and had limited statistical precision.

## Introduction

Breast lesions constitute one of the most common diagnostic workloads in surgical pathology and cytopathology, spanning inflammatory, benign proliferative, atypical and malignant entities. Breast cancer remains the most frequently diagnosed cancer among women worldwide, and GLOBOCAN 2022 estimates continue to show a major disease burden. In India, breast cancer has overtaken cervical cancer in several registries as the leading female malignancy, creating a persistent need for accessible early diagnostic pathways [1,2].

Fine-needle aspiration cytology (FNAC) remains practically relevant because it is inexpensive, repeatable, minimally invasive and capable of providing rapid preliminary categorization of palpable and image-detected breast lesions. Although core needle biopsy (CNB) is indispensable for tissue architecture, invasion assessment and receptor profiling, Yokohama-guided FNAC can bridge much of the initial diagnostic accuracy gap between FNAC and CNB by converting cytology into a standardized triage tool with fewer procedural demands and lower morbidity. Its diagnostic value is highest when integrated into the triple assessment model combining clinical examination, radiological imaging and pathological evaluation. However, traditional descriptive FNAC reporting has been limited by variable terminology and inconsistent communication of borderline categories [3-7].

The International Academy of Cytology (IAC) Yokohama System for Reporting Breast Fine-Needle Aspiration Biopsy Cytopathology was introduced to address this limitation. The system classifies breast aspirates into five categories: C1 inadequate/insufficient, C2 benign, C3 atypical probably benign, C4 suspicious for malignancy and C5 malignant. Each category is linked to an expected risk of malignancy (ROM) and broad management implications, thereby allowing cytology reports to function as clinically actionable risk-stratification tools [8,9].

Several institutional studies and pooled analyses have reported high sensitivity and specificity for the Yokohama System, and recent work has also emphasized the importance of concordance between IAC Yokohama cytology and Breast Imaging Reporting and Data System (BI-RADS) imaging categories. However, category distribution and ROM vary by setting, sampling practice, cytopathologist experience and histopathological follow-up patterns [10-12]. Local validation is therefore necessary before routine adoption. The present study evaluates the distribution, ROM, BI-RADS-cytology concordance and diagnostic accuracy of the IAC Yokohama System in breast FNAC cases from a tertiary care teaching hospital in Central India.

The primary objective was to categorize breast fine-needle aspirates using the IAC Yokohama System and evaluate its diagnostic accuracy through histopathological correlation. A secondary objective was to compare two clinically relevant diagnostic thresholds: Scenario A, a high-sensitivity model in which C2 was classified as negative and C3-C5 as positive, and Scenario B, an actionable-positive model in which C2-C3 were classified as negative and C4-C5 as positive.

## Materials and methods

Study design and reporting framework

**Figure 1:**
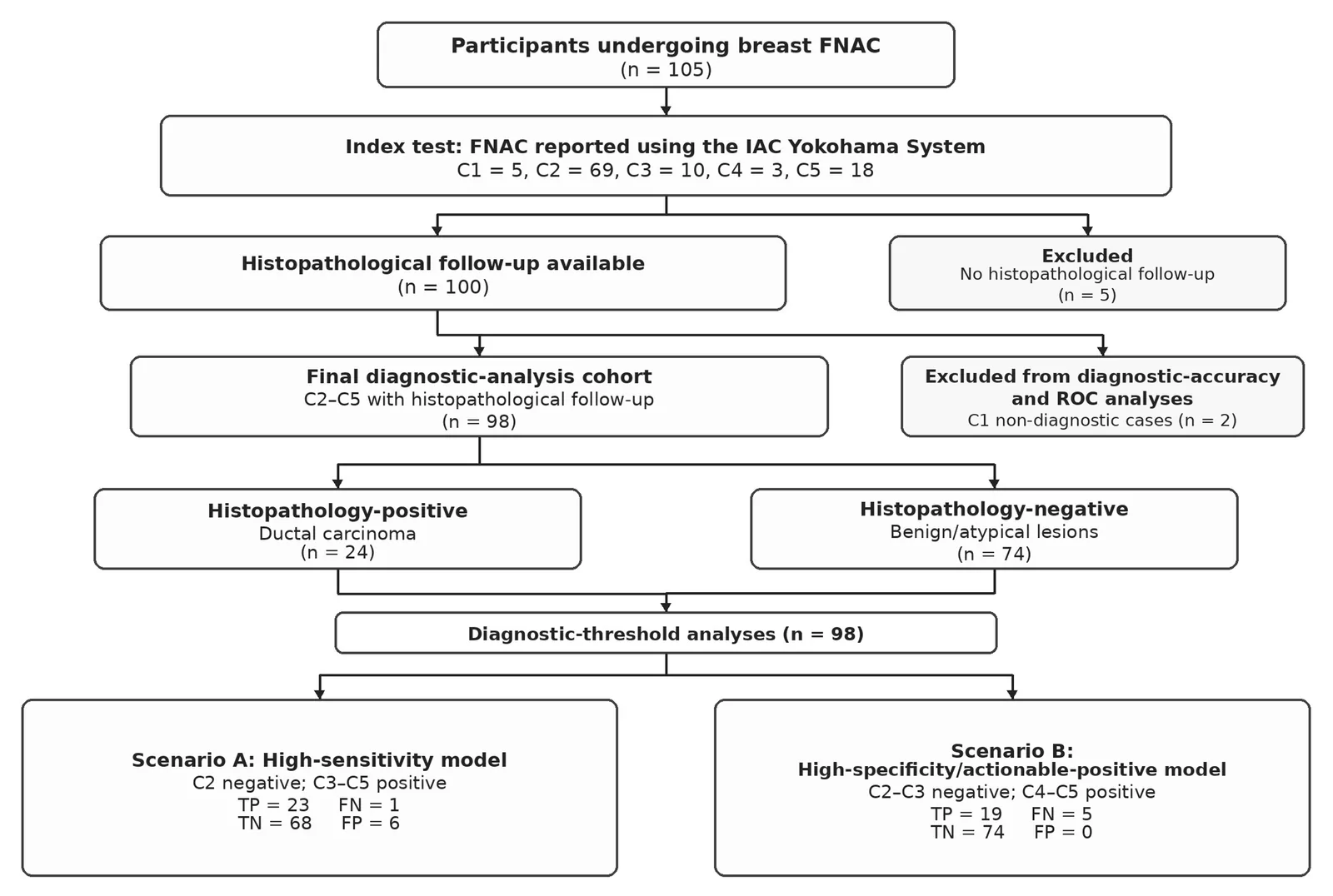
Participant flow and diagnostic classification of breast FNAC cases Participant flow diagram showing enrolment, IAC Yokohama categorization, histopathological follow-up, exclusion of non-diagnostic C1 cases, and the final diagnostic-analysis cohort. Of 105 participants, histopathological follow-up was available for 100 cases. Two C1 non-diagnostic cases were excluded from diagnostic-accuracy and ROC analyses, leaving 98 cases categorized as C2-C5. Scenario A classified C2 as cytology-negative and C3-C5 as cytology-positive. Scenario B classified C2-C3 as cytology-negative and C4-C5 as cytology-positive. FNAC, fine-needle aspiration cytology; IAC, International Academy of Cytology; ROC, receiver operating characteristic.

This observational diagnostic accuracy study was structured according to the Standards for Reporting Diagnostic Accuracy Studies (STARD) 2015 statement [13]. The index test was breast FNAC categorized by the IAC Yokohama System, and histopathology from biopsy or excision specimens was used as the reference standard wherever available. Participant flow through index testing, histopathological follow-up and diagnostic accuracy analysis is shown in Figure 1.

Study setting

The study was conducted from May 2024 to October 2025 in the Department of Pathology, Gandhi Medical College and Hamidia Hospital, Bhopal, Madhya Pradesh, India.

Participants and eligibility criteria

All patients advised to undergo breast FNAC during the study period were considered eligible. Inclusion criteria were patients undergoing breast FNAC and willing to participate after informed consent. Exclusion criteria were previously diagnosed breast malignancy and patients already receiving chemotherapy or radiotherapy. Availability of histopathological follow-up was not a requirement for inclusion in the overall FNAC cohort. Cases without histopathological follow-up were retained for the descriptive analysis but were excluded from the diagnostic accuracy analysis.

Sampling and sample size

A total of 105 patients were included. The sample size was determined by institutional feasibility and case availability during the study period and was considered adequate for estimating diagnostic accuracy in a single-center validation cohort with 100 histopathologically correlated cases.

FNAC procedure and cytological processing

FNAC was performed under aseptic precautions using a 21-25 gauge needle attached to a 10-20 mL disposable syringe. Palpation guidance was used for clinically palpable lesions, while ultrasound guidance was used for non-palpable or deep-seated lesions when required. Aspirated material was smeared on clean glass slides and processed using routine cytological stains including haematoxylin and eosin, Papanicolaou and Giemsa stains as per standard laboratory protocols [14-16].

Index test and reference standard

Aspirates were categorized according to the IAC Yokohama System into C1 inadequate/insufficient, C2 benign, C3 atypical probably benign, C4 suspicious for malignancy and C5 malignant [8,9]. Histopathological diagnosis from biopsy or excision specimens was considered the reference standard. Core biopsy or surgical excision was undertaken independently as part of routine clinical management, based on documented clinical and radiological indications, therapeutic considerations, and decisions made by the treating surgeon and patient. The study used the histopathological diagnoses generated through these routine procedures for subsequent cytohistological correlation. Formal blinding of histopathology assessment to the prior FNAC Yokohama category was not documented in the study dataset; therefore, the reference-standard interpretation should be considered part of the routine clinical diagnostic workflow rather than a prospectively blinded review protocol. This point was retained transparently as a methodological limitation.

Outcome measures and statistical analysis

The primary outcomes were category-wise distribution, ROM and diagnostic accuracy. ROM was calculated as the number of histopathologically confirmed carcinomas in a Yokohama category divided by the number of cases with available histopathology in that category, multiplied by 100. Histopathologically confirmed carcinoma was classified as a positive reference-standard outcome, whereas benign and atypical histopathological lesions were classified as non-malignant outcomes.

C1 cases were excluded from diagnostic-accuracy analyses because C1 represents a non-diagnostic result rather than a negative cytological diagnosis. Two diagnostic thresholds were evaluated among cases categorized as C2-C5. Scenario A, the high-sensitivity model, classified C2 as cytology-negative and C3-C5 as cytology-positive. Scenario B, the high-specificity/actionable-positive model, classified C2-C3 as cytology-negative and C4-C5 as cytology-positive.

Sensitivity, specificity, positive predictive value (PPV), negative predictive value (NPV) and overall diagnostic accuracy were calculated from the corresponding 2 × 2 contingency tables. Two-sided 95% confidence intervals for these diagnostic indices and category-specific ROM estimates were calculated using the Clopper-Pearson exact binomial method. ROC analysis was performed using the ordinal Yokohama categories, and the area under the curve (AUC) with its 95% confidence interval was estimated using the DeLong method.

Descriptive statistics, cross-tabulations and Pearson’s chi-square tests were performed using IBM SPSS Statistics version 25.0 (IBM Corp., Armonk, NY, USA). Diagnostic estimates and confidence intervals were verified using jamovi version 2.6.44 (The jamovi project, Sydney, Australia). Python (Python Software Foundation, Wilmington, DE) was used for ROC analysis and visualization. A two-sided p-value <0.05 was considered statistically significant.

Ethical considerations

Institutional Ethics Committee approval was obtained from Gandhi Medical College and Hamidia Hospital, Bhopal, before data collection. Written informed consent was obtained from all participants. Patient confidentiality was maintained, and the procedure involved no therapeutic intervention beyond routine diagnostic FNAC.

## Results

Baseline clinical and imaging profile

The study included 105 breast FNAC cases. The cohort showed a strong female predominance, with 99 female cases (94.3%) and six male cases (5.7%). Most participants were married (75.2%). Lactational history was absent in 100 cases (95.2%), and addiction history was absent in 101 cases (96.2%). Ultrasonography (USG) showed BI-RADS III as the most frequent imaging category, followed by BI-RADS IV. The baseline and imaging profile are summarized in Table 1, and the BI-RADS-Yokohama cross-tabulation is shown in Table 2.

**Table 1:** Baseline demographic, clinical and imaging profile of study participants (n = 105) BI-RADS, Breast Imaging Reporting and Data System.

Variable	Category	Frequency (%)
Gender	Female	99 (94.3)
	Male	6 (5.7)
Marital status	Married	79 (75.2)
	Unmarried	26 (24.8)
Lactational history	Absent	100 (95.2)
	Present	5 (4.8)
Addiction history	Absent	101 (96.2)
	Tobacco use	2 (1.9)
	Smoking	1 (1.0)
BI-RADS category	BI-RADS I	12 (11.4)
	BI-RADS II	17 (16.2)
	BI-RADS III	46 (43.8)
	BI-RADS IV	26 (24.8)
	BI-RADS V	4 (3.8)

**Table 2:** Cross-tabulation of ultrasonography BI-RADS categories and IAC Yokohama cytology categories in 105 breast FNAC cases BI-RADS, Breast Imaging Reporting and Data System; IAC, International Academy of Cytology; FNAC, fine-needle aspiration cytology.

BI-RADS category	C1	C2	C3	C4	C5	Total
BI-RADS I	1	10	1	0	0	12
BI-RADS II	2	14	1	0	0	17
BI-RADS III	1	39	3	1	2	46
BI-RADS IV	1	6	5	2	12	26
BI-RADS V	0	0	0	0	4	4
Total	5	69	10	3	18	105

Cross-tabulation showed a meaningful radiology-cytology gradient. Among 30 BI-RADS IV/V cases, 18 (60.0%) were classified into the high-specificity/actionable cytology categories C4-C5, while 23 (76.7%) were classified into Scenario A positive categories C3-C5. All BI-RADS V cases were captured by malignant cytology (C5, 4/4; 100%). Conversely, 67 of 75 BI-RADS I-III cases (89.3%) fell within C1-C2, supporting multidisciplinary concordance between imaging suspicion and Yokohama cytological risk stratification.

Yokohama cytology and histopathology distribution

Using the IAC Yokohama dataset, most aspirates were categorized as C2 benign (65.7%), followed by C5 malignant (17.1%), C3 atypical (9.5%), C1 inadequate (4.8%) and C4 suspicious for malignancy (2.9%). Histopathology showed 72 benign lesions (68.6%), 24 ductal carcinomas (22.9%), four atypical/intermediate lesions (3.8%), and no available histopathology in five cases (4.8%). The four atypical/intermediate lesions comprised two cases of atypical ductal hyperplasia, one atypical fibroadenoma and one borderline phyllodes tumour. The cytology and histopathology distributions are presented in Table 3.

**Table 3:** Distribution of Yokohama cytological categories and histopathological diagnoses Atypical/intermediate lesions comprised two cases of atypical ductal hyperplasia, one atypical fibroadenoma, and one borderline phyllodes tumour.

Diagnostic domain	Category	Frequency (%)
Yokohama cytology	C1 Inadequate	5 (4.8)
	C2 Benign	69 (65.7)
	C3 Atypical	10 (9.5)
	C4 Suspicious for malignancy	3 (2.9)
	C5 Malignant	18 (17.1)
Histopathology	Benign lesions	72 (68.6)
	Ductal carcinomas	24 (22.9)
	Atypical lesions	4 (3.8)
	Not available	5 (4.8)

Cyto-histopathological correlation and ROM

Histopathology was available for 100 cases, but the two C1 cases were excluded from diagnostic accuracy. Histopathological follow-up was available for all 69 C2 cases because these patients proceeded to core biopsy or surgical excision during routine clinical management. The indications included clinical-radiological discordance (n = 6), persistent or enlarging lesions (n = 18), symptomatic lesions or therapeutic excision (n = 21), and patient or treating-surgeon preference (n = 24). Among C2 cases, 67 were benign, one was atypical and one was carcinoma, yielding a ROM of 1.4% (95% CI 0.04-7.8). In C3, three cases were benign, three were atypical and four were carcinoma, producing a ROM of 40.0% (95% CI 12.2-73.8). All three C4 cases were carcinoma, corresponding to a ROM of 100.0% (95% CI 29.2-100.0). All 16 C5 cases with histopathology were carcinoma, corresponding to a ROM of 100.0% (95% CI 79.4-100.0). The wide confidence intervals for C1 and C4 reflect the small numbers of cases in these categories. Category-wise histopathological distribution and ROM are shown in Table 4.

**Table 4:** Histopathological distribution and risk of malignancy across IAC Yokohama categories CI, confidence interval; ROM, risk of malignancy; IAC, International Academy of Cytology. Two-sided 95% CIs were calculated using the Clopper-Pearson exact binomial method.

Yokohama category	Total cases with histopathology	Atypical lesions n (%)	Benign lesions n (%)	Ductal carcinoma n (%)	ROM, % (95% CI)
C1 Inadequate	2	0 (0.0)	2 (100.0)	0 (0.0)	0.0 (0.0-84.2)
C2 Benign	69	1 (1.4)	67 (97.1)	1 (1.4)	1.4 (0.04-7.8)
C3 Atypical	10	3 (30.0)	3 (30.0)	4 (40.0)	40.0 (12.2-73.8)
C4 Suspicious	3	0 (0.0)	0 (0.0)	3 (100.0)	100.0 (29.2-100.0)
C5 Malignant	16	0 (0.0)	0 (0.0)	16 (100.0)	100.0 (79.4-100.0)

**Figure 2:**
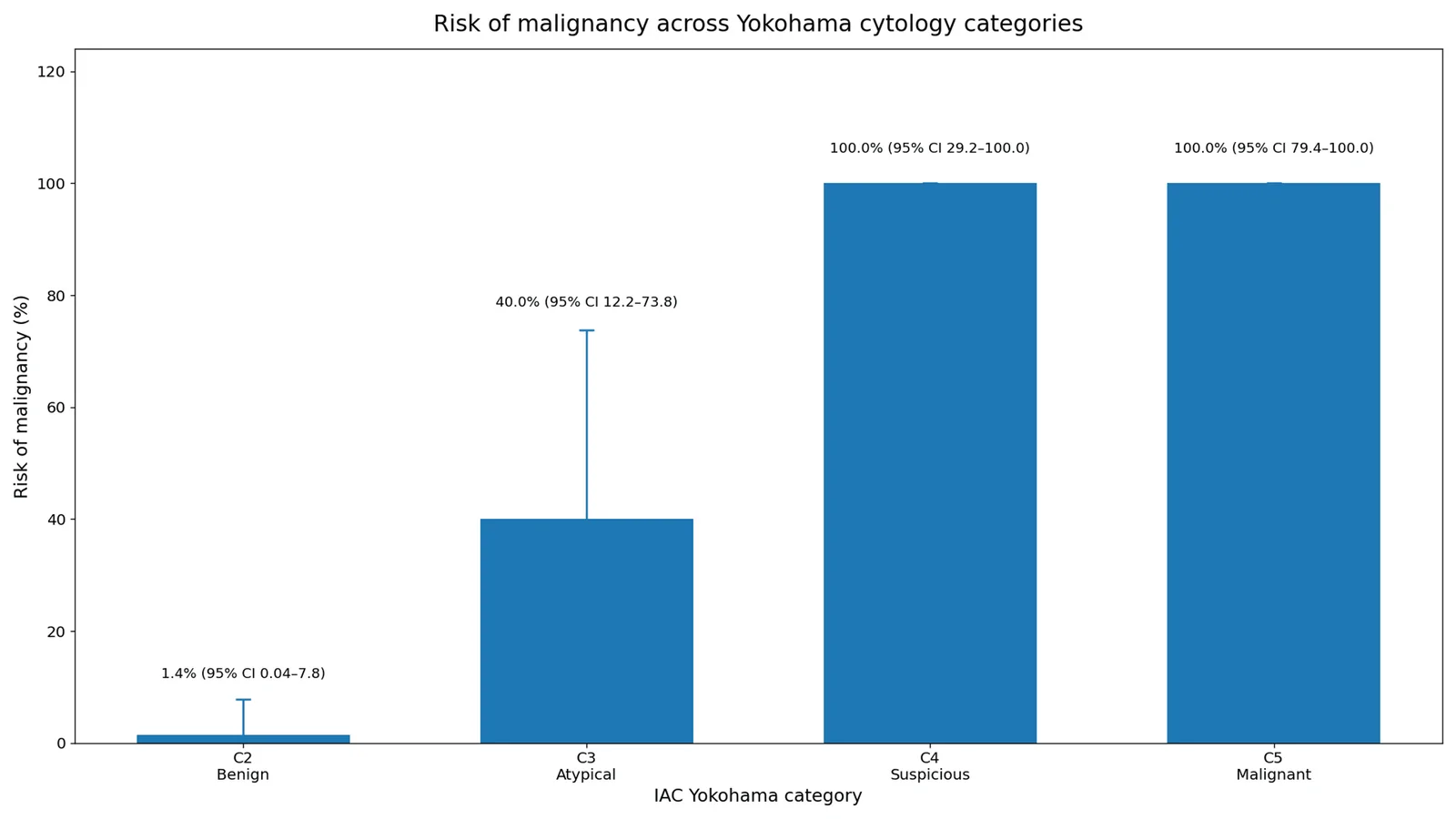
Risk of malignancy across IAC Yokohama categories CI, confidence interval; IAC, International Academy of Cytology; ROM, risk of malignancy.

A progressive rise in ROM was observed from the lower to higher Yokohama categories, with the main diagnostic uncertainty concentrated in the C3 atypical group. The ROM gradient is illustrated in Figure 2.

Diagnostic performance

**Figure 3:**
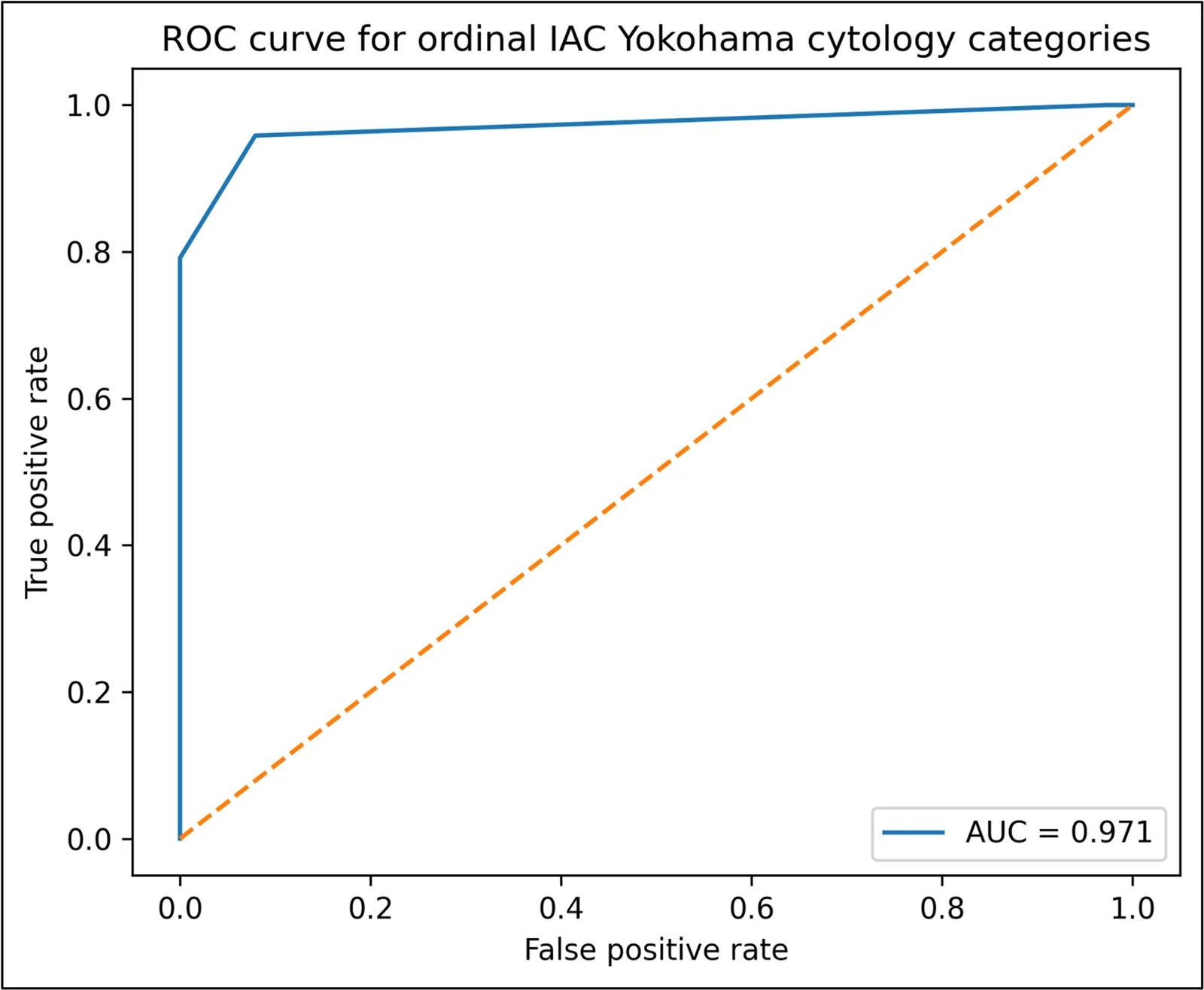
ROC curve for ordinal IAC Yokohama cytology categories Receiver operating characteristic curve for ordinal IAC Yokohama cytology categories against histopathology. AUC = 0.971 (95% CI 0.926-1.000). AUC, area under the curve; IAC, International Academy of Cytology; ROC, receiver operating characteristic.

Diagnostic-performance analyses were restricted to the 98 cases categorized as C2-C5 with histopathological follow-up. The two C1 non-diagnostic cases were excluded. Scenario A (high-sensitivity model; C3-C5 positive) showed 23 true positives, 70 true negatives, six false positives and one false negative, producing sensitivity of 95.8%, specificity of 92.1%, PPV of 79.3%, NPV of 98.6%, overall accuracy of 93.0% and ROC-AUC of 0.971. Scenario B (high-specificity/actionable-positive model; C4-C5 positive) showed 19 true positives, 76 true negatives, no false positives and five false negatives, producing sensitivity of 79.2%, specificity of 100.0%, PPV of 100.0%, NPV of 93.8% and accuracy of 95.0%. The cytology-histopathology association was statistically significant in Scenario A (Pearson chi-square = 68.506, df = 1, p < 0.001). ROC analysis using ordinal Yokohama category scores yielded an AUC of 0.971, indicating excellent discrimination. Diagnostic indices are summarized in Table 5, and the ROC curve is shown in Figure 3.

**Table 5:** Diagnostic correlation and performance of the IAC Yokohama System against histopathology using Scenario A and Scenario B diagnostic thresholds IAC, International Academy of Cytology. Values inside parentheses represent the confidence intervals. Diagnostic-performance confidence intervals were calculated using the two-sided Clopper-Pearson exact method.

Diagnostic parameter	Scenario A: High-sensitivity model (C3-C5 positive)	Scenario B: High-specificity/actionable-positive model (C4-C5 positive)
True positive	23	19
True negative	68	74
False positive	6	0
False negative	1	5
Sensitivity	95.8 (78.9-99.9)	79.2 (57.8-92.9)
Specificity	91.9 (83.2-97.0)	100.0 (95.3-100.0)
Positive predictive value	79.3 (60.3-92.0)	100.0 (82.4-100.0)
Negative predictive value	98.6 (92.4-100.0)	93.8 (86.2-98.0)
Overall diagnostic accuracy	93.0 (86.1-97.1)	95.0 (88.7-98.4)
Pearson chi-square	66.938	72.673
p-Value	<0.001	<0.001

The ROC curve based on ordinal Yokohama scores demonstrated near-complete separation between histopathologically malignant and non-malignant cases, with an AUC of 0.971 (Figure 3).

Grey-zone category and discordant cases

The C3 atypical category accounted for the six false-positive cases in the primary diagnostic threshold: three were benign lesions and three were atypical lesions on histopathology. The four malignant outcomes within C3 belonged to the ductal carcinoma/ductal carcinoma in situ (DCIS) spectrum, including cases recorded as invasive carcinoma of no special type, infiltrating ductal carcinoma, intraductal papilloma with DCIS, and borderline phyllodes tumour with foci of DCIS. One false-negative case was observed in the C2 benign category, corresponding to a C2 ROM of 1.4%. This reinforces the need for clinical and radiological correlation even when cytology appears benign.

Representative cytomorphology

**Figure 4:**
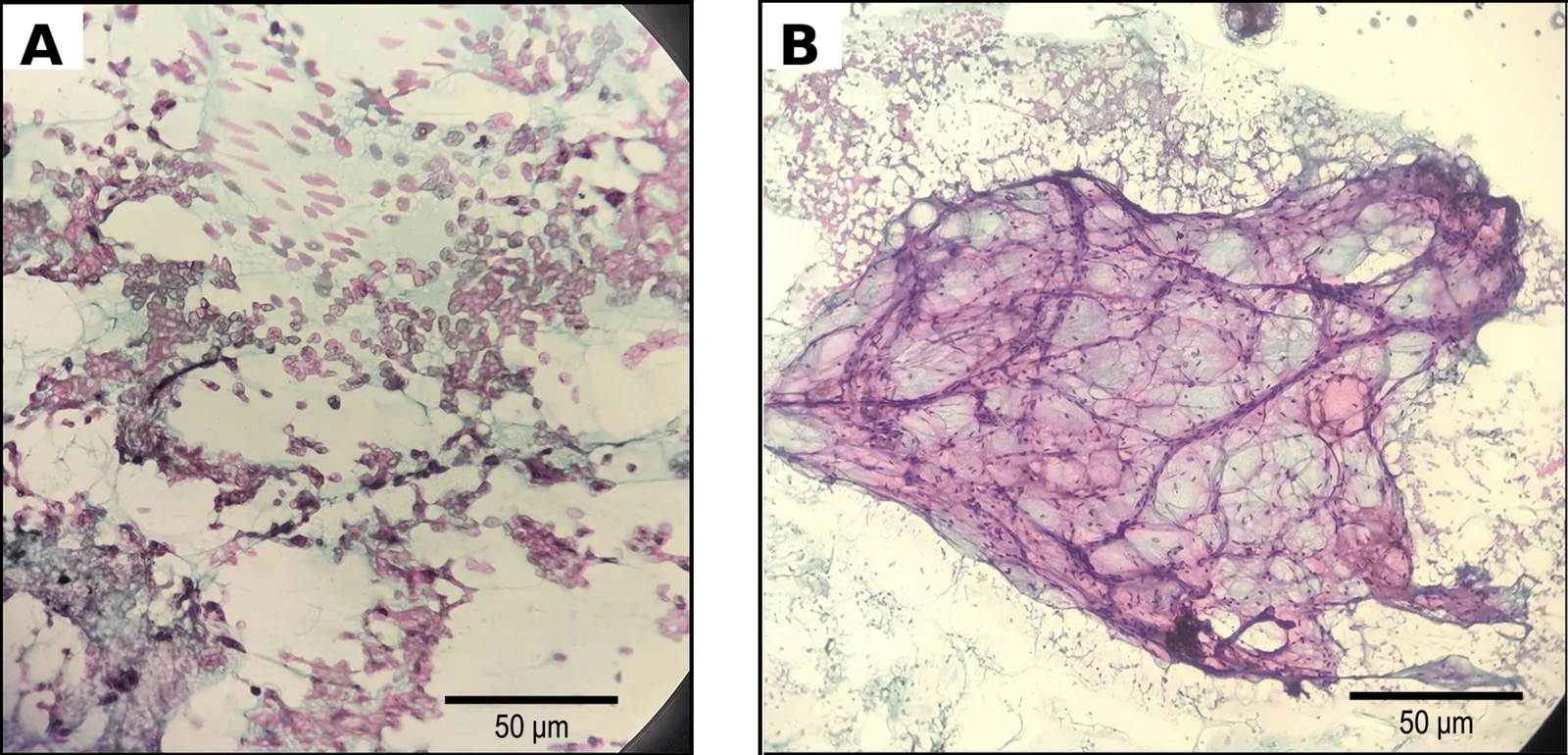
Representative IAC Yokohama Category I/inadequate smears (A) Haemorrhagic material inadequate for interpretation. (B) Smear showing mature adipocytes without identifiable duct epithelial cells. Stain and magnification: (A) Papanicolaou stain, 400×; (B) Papanicolaou stain, 400×. IAC, International Academy of Cytology.

**Figure 5:**
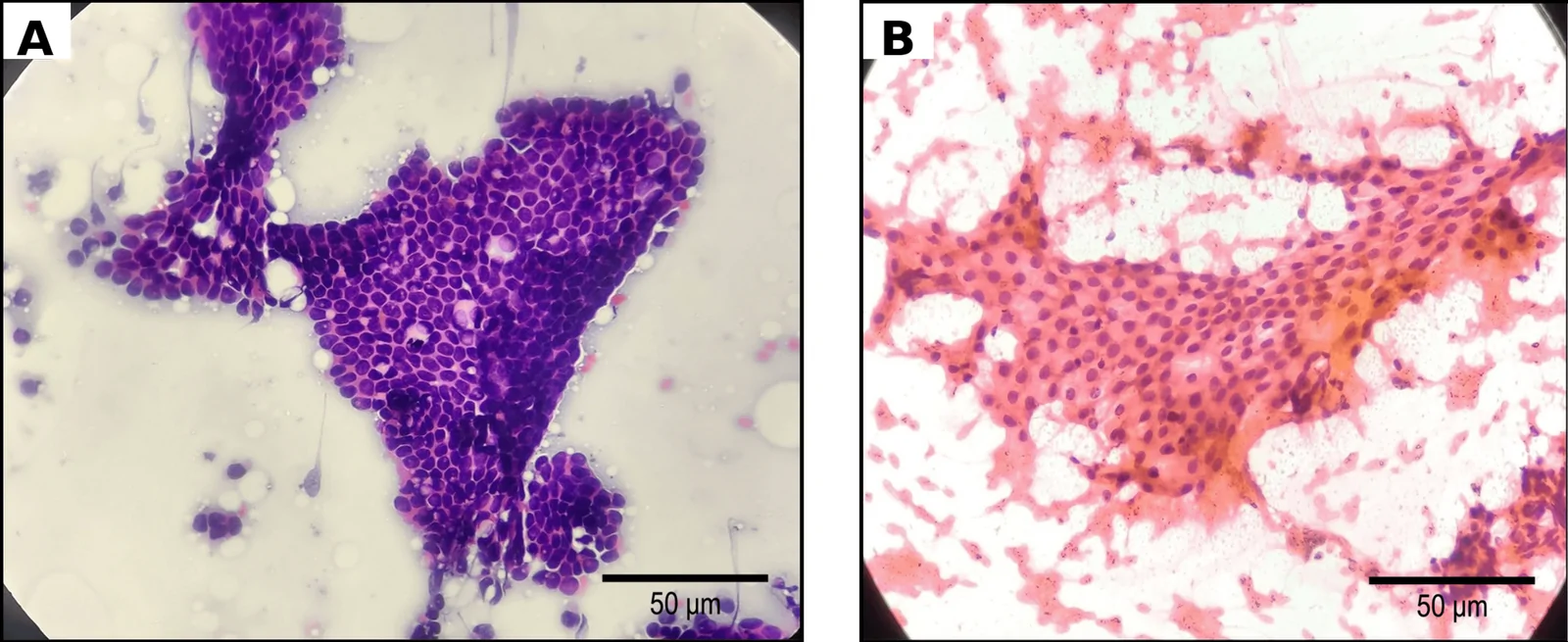
Representative Category II/benign smears (A) Cohesive sheets of monomorphic ductal epithelial cells with overlying myoepithelial nuclei. (B) Numerous bare bipolar myoepithelial nuclei in the background, supporting a benign biphasic cytological pattern. Stain and magnification: (A) Haematoxylin and eosin stain, 400×; (B) Papanicolaou stain, 400×.

**Figure 6:**
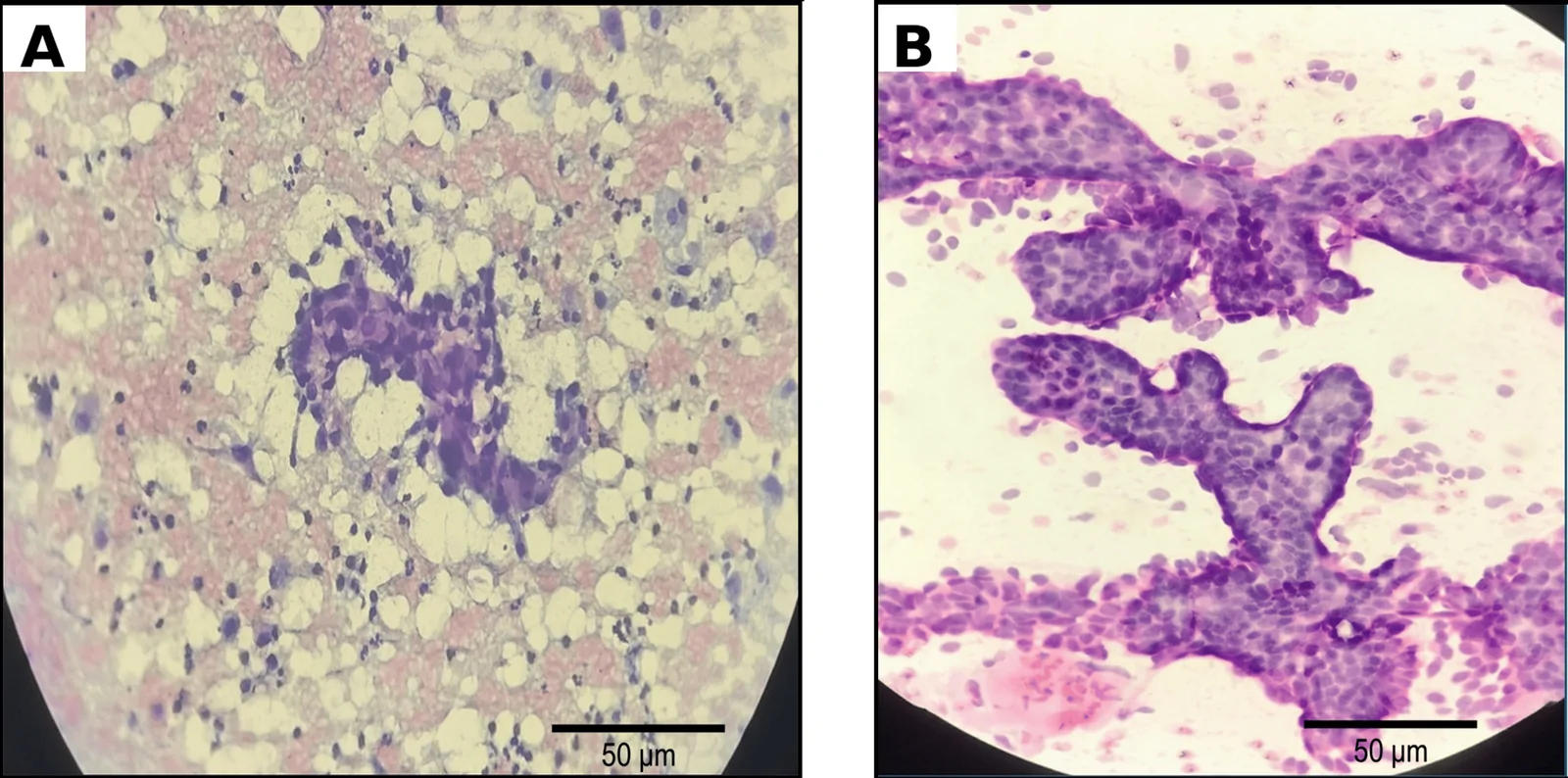
Representative Category III/atypical smears (A) Few atypical ductal cells with nuclear pleomorphism and hyperchromasia. (B) Focal epithelial proliferation in a papillary-like pattern. Stain and magnification: (A) Haematoxylin and eosin stain, 400×; (B) Haematoxylin and eosin stain, 400×.

**Figure 7:**
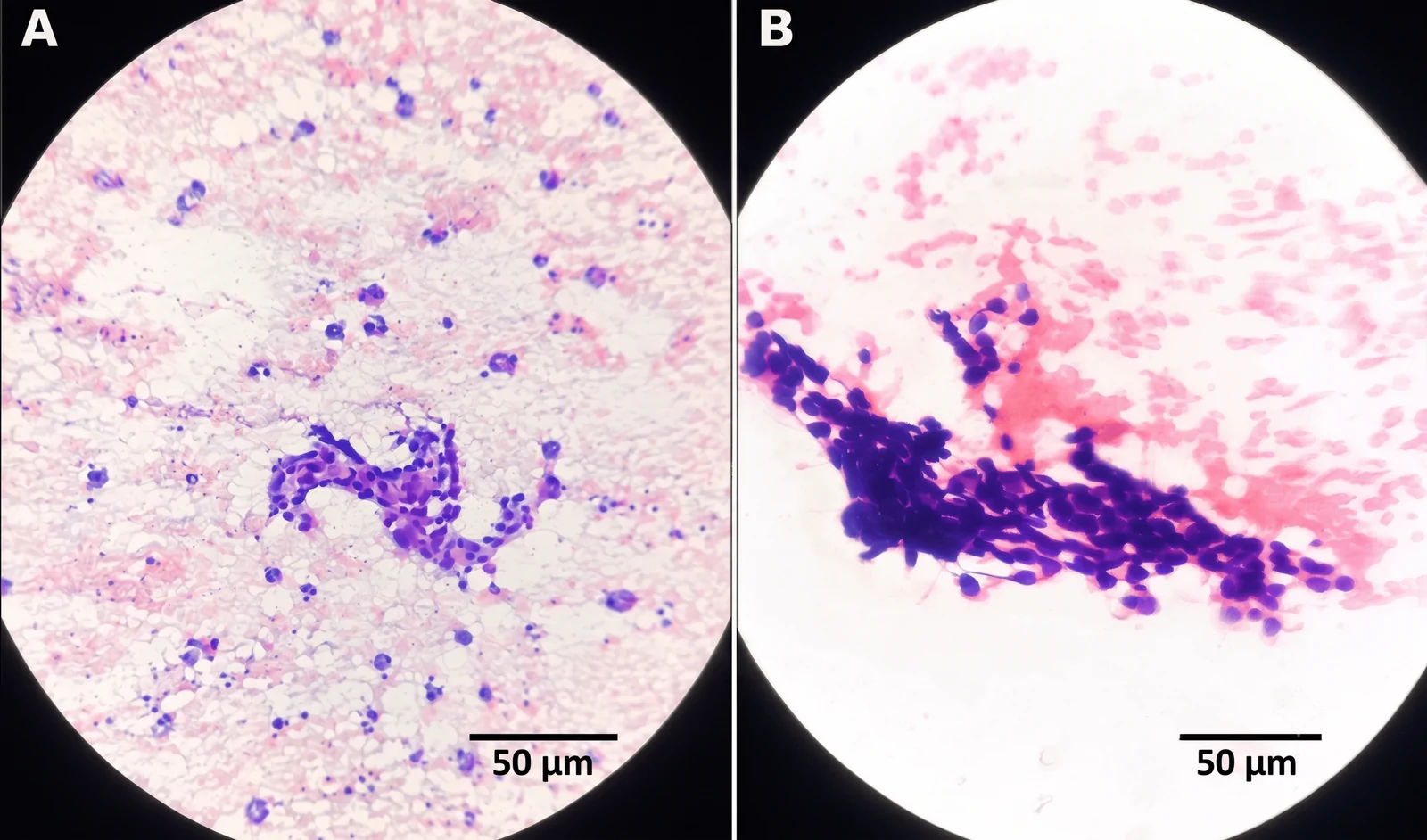
Representative Category IV/suspicious for malignancy smears (A) Sheets of ductal cells with nuclear crowding and overlapping. (B) Ductal cells showing pleomorphism, overlap and hyperchromasia. Stain and magnification: (A) Papanicolaou stain, 400×; (B) Haematoxylin and eosin stain, 400×.

**Figure 8:**
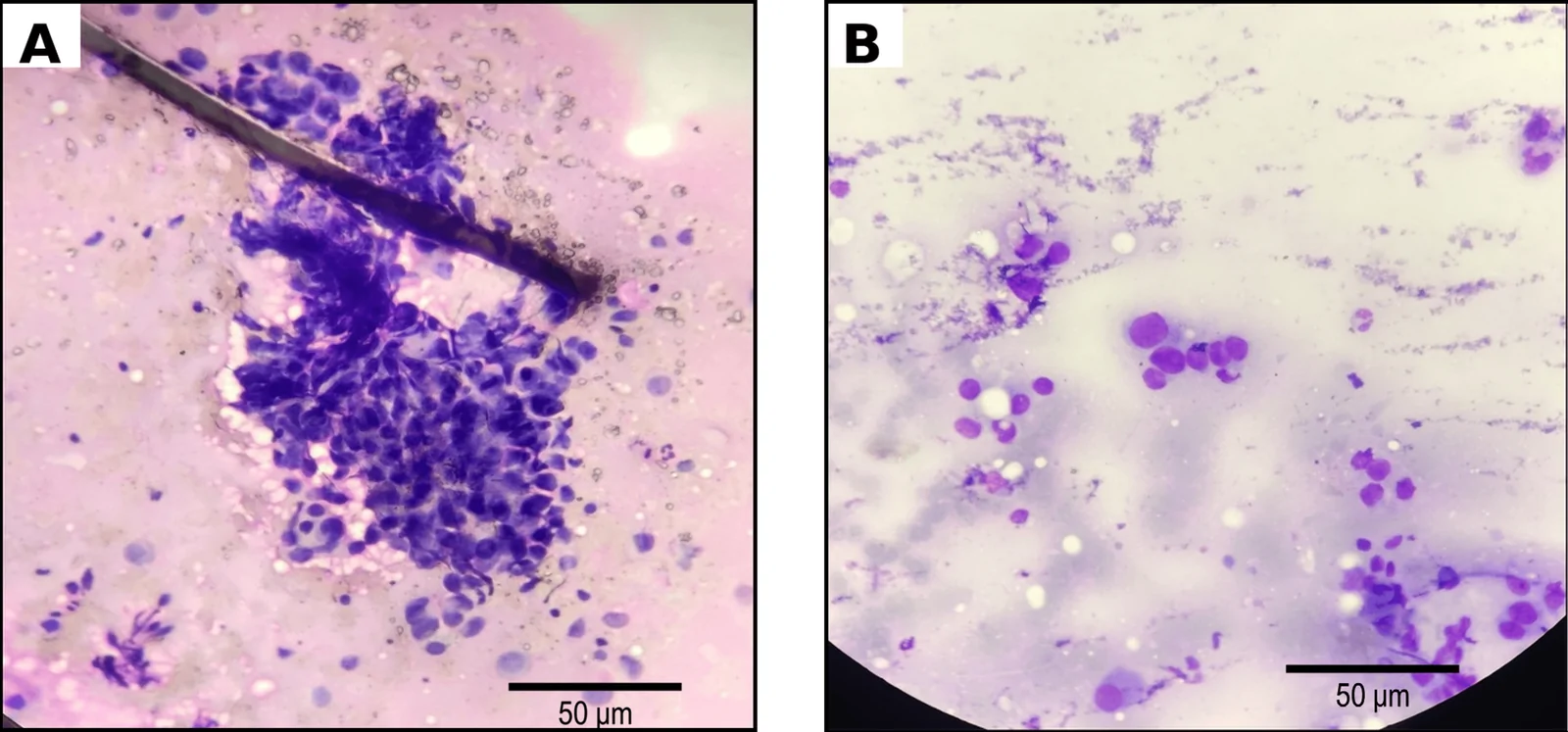
Representative Category V/malignant smears (A) Highly pleomorphic tumour cells with marked atypia. (B) Tumour cells with high nucleocytoplasmic ratio and coarse chromatin. Stain and magnification: (A) Haematoxylin and eosin stain, 400×; (B) Giemsa stain, 400×.

Representative original cytology photomicrographs for the five Yokohama categories are shown in Figures 4-8.

## Discussion

This study supports the clinical utility of the IAC Yokohama System as a standardized framework for reporting breast FNAC in a tertiary care setting. The dominant cytological category was C2 benign, which is expected in breast lump clinics where a large proportion of lesions are fibroadenoma, fibrocystic change, inflammatory conditions or other benign processes. More importantly, ROM increased in a biologically and clinically coherent manner from C2 through C5, demonstrating the value of the reporting system as a risk-stratification tool rather than a mere descriptive classification.

The observed ROM for C2 was 1.4% (1/69). The C2 cases with tissue follow-up represent lesions that proceeded to biopsy or excision for independent clinical, radiological or therapeutic indications. Therefore, the 100% histopathological follow-up rate in this category should not be interpreted as a recommendation for routine biopsy for all C2 lesions. When the clinical, radiological and cytological findings are concordantly benign, additional tissue sampling is generally not required.

The C3 atypical category had a ROM of 40% in the present cohort. This is the most important practical finding because it shows why the atypical category must not be treated as benign. Atypical aspirates represent a cytological threshold where proliferative benign lesions, papillary lesions, atypical ductal proliferations, DCIS and low-grade carcinomas may overlap. Published Yokohama literature similarly identifies C3 as the most variable and management-sensitive category [17-27]. In practice, C3 should trigger careful clinical-radiological correlation and tissue diagnosis rather than reassurance.

The higher Yokohama categories showed high observed ROM, but the precision of the estimates differed substantially. C4 had a ROM of 100.0% based on only three cases, with a wide exact 95% CI of 29.2-100.0%, whereas C5 had a ROM of 100.0% based on 16 cases, with a 95% CI of 79.4-100.0%. Consequently, the absence of a non-malignant outcome among the three C4 cases should not be interpreted as establishing a universally 100% ROM for this category. Similarly, the observed 100% specificity and PPV under Scenario B retained sampling uncertainty, with lower confidence limits of 95.3% and 82.4%, respectively. Nevertheless, C4 and C5 remain clinically actionable categories requiring prompt tissue confirmation and appropriate surgical or oncological evaluation.

This positioning is consistent with the current FNAC-CNB literature: CNB remains necessary when architecture and biomarker profiling are needed, but standardized FNAC reporting can function as a rapid, lower-risk first-line triage method. In this sense, the Yokohama System does not compete with CNB; it bridges the practical accuracy gap by identifying which cases can be confidently reassured, which require biopsy, and which need immediate oncological workup [6,7,11].

The present BI-RADS-Yokohama cross-tabulation also strengthens the multidisciplinary relevance of the study. A majority of BI-RADS IV/V cases moved into Scenario A-positive cytology categories, and all BI-RADS V lesions were assigned to C5. This supports the view that BI-RADS and Yokohama reporting provide complementary languages for radiology-pathology communication rather than isolated diagnostic silos [12].

C1 was excluded from diagnostic-performance and ordinal ROC analyses because inadequate cytology does not constitute a negative diagnostic result and does not represent a defined position on the biological malignancy-risk continuum. In the corrected analysis restricted to C2-C5 cases, Scenario A classified C2 as cytology-negative and C3-C5 as cytology-positive. Sensitivity was 95.8% (95% CI 78.9-99.9), specificity was 91.9% (95% CI 83.2-97.0), PPV was 79.3% (95% CI 60.3-92.0), NPV was 98.6% (95% CI 92.2-100.0), and overall accuracy was 92.9% (95% CI 85.8-97.1). The high NPV therefore applies to C2 as the negative cytological category and should not be interpreted as evidence that a C1 non-diagnostic result is reassuring. The lower PPV reflects the inclusion of C3 with C4 and C5 as cytology-positive, because not all atypical lesions were malignant. Other studies have reported high diagnostic indices for the Yokohama System, including the studies by Sah et al. [17], Montezuma et al. [18], Agrawal et al. [19], Niaz et al. [23], Dixit et al. [24], Khan et al. [25], Ravikumar et al. [26], Verma et al. [28], and Ahuja and Malviya [29].

The findings also align with the systematic review by Paul et al. [20] and the meta-analysis by Nikas et al. [11], which support the overall diagnostic strength of the IAC Yokohama System in breast FNAC. Compared with unstructured reporting, the principal advantage of Yokohama terminology is that it gives clinicians a defined risk language. This matters in high-volume Indian tertiary care settings, where quick and cost-effective triage is essential and where communication gaps between pathology, radiology and surgery can delay management. The findings compare favorably with published Yokohama validation studies and meta-analyses (Table 6).

**Table 6:** Comparison of the present study with selected published studies using the Yokohama System NR, not reported; PPV, positive predictive value; NPV, negative predictive value.

Study	Design/sample	Positive cytology threshold	Sensitivity (%)	Specificity (%)	PPV (%)	NPV (%)	Accuracy (%)	Key comparison
Montezuma et al. (2019) [18]	Single-institution retrospective series; 3,625 FNAB samples; 776 with histopathology	Malignant category positive	97.56	100	NR	NR	99.11	Very high specificity and accuracy; lower category threshold differs from present study.
Ahuja and Malviya (2021) [29]	Retrospective tertiary-care series; 554 aspirates; 242 with histopathology	Atypical, suspicious and malignant positive	97.20	86.00	77.00	98.50	89.60	Similar high-sensitivity approach; present study had higher specificity and accuracy.
Niaz et al. (2022) [23]	Institutional retrospective series; 2,133 aspirates; 421 with histopathology	Absolute malignant sensitivity/specificity model	83.42	85.24	NR	NR	NR	Lower sensitivity and specificity; reported higher C1 and C2 ROM than many series.
Yadav et al. (2024) [12]	Retrospective tertiary-care series; 762 aspirates; 321 with histopathology	Atypical, suspicious and malignant positive	95.78	90.29	81.98	97.90	86.29	Sensitivity similar to present study; present accuracy was higher.
Verma et al. (2024) [28]	Central India tertiary-care series; 216 aspirates; 144 with histopathology	Atypical considered positive	87.50	100	100	93.50	95.56	Higher specificity; present study had higher sensitivity and lower specificity.
Present study	105 aspirates; 100 with histopathology; 98 included in diagnostic-performance analysis	Atypical, suspicious and malignant positive	95.80	91.9	79.30	98.60	93.00	Strong sensitivity and NPV; the single false-negative case occurred in C2.

The study has direct institutional relevance. It demonstrates that routine FNAC, when reported using a validated five-tier system, can achieve strong diagnostic performance even without extensive ancillary testing. At the same time, the data expose the limits of FNAC: inadequate aspirates, atypical lesions, interpretative overlap in the C3 category and the absence of a documented prospective blinding protocol remain important limitations. Therefore, the Yokohama System should be adopted as part of a multidisciplinary diagnostic pathway rather than used as a standalone replacement for histopathology.

Limitations

This study has limitations. First, it was conducted at a single tertiary care centre and may reflect local referral patterns, case mix and cytopathology expertise. Second, the overall sample size was modest, and some Yokohama categories contained very few cases, particularly C1 and C4. Consequently, category-specific ROM estimates had wide confidence intervals. The observed C4 ROM of 100.0% was based on only three cases and had an exact 95% CI of 29.2-100.0%; it should therefore be interpreted as an imprecise cohort-specific estimate rather than a definitive risk value. Similarly, the apparently perfect specificity and PPV under Scenario B retained statistical uncertainty. Third, histopathology was unavailable in five cases, although the diagnostic-accuracy analysis was restricted to cases with tissue follow-up. The unusually high histopathological verification rate among C2 cases may have introduced work-up or verification bias and spectrum bias, because lesions undergoing biopsy or excision may not represent all C2 cases managed through clinicoradiological follow-up. Therefore, the observed C2 false-negative rate, ROM, specificity and NPV may not be fully generalizable to routine C2 populations. Larger prospective multicentre studies with blinded histopathological review, interobserver analysis and structured triple assessment are required to obtain more stable category-specific and diagnostic-performance estimates.

## Conclusions

The IAC Yokohama System provides a structured and clinically useful framework for reporting breast FNAC by converting cytological findings into standardized diagnostic categories with practical management relevance. Its use can improve communication between pathologists, radiologists and clinicians, particularly when breast FNAC is interpreted as part of triple assessment rather than as an isolated diagnostic test.

The atypical category remains the main interpretative grey zone and should prompt careful clinicoradiological correlation and histopathological confirmation. Higher-risk Yokohama categories can support timely surgical or oncological evaluation in clinically concordant cases. In resource-limited settings, standardized FNAC reporting may help provide rapid and cost-effective diagnostic triage while maintaining the need for tissue confirmation where architecture, invasion status or ancillary testing is required.
